# Systemic Therapy for Advanced Hepatocellular Carcinoma in 2026: Current Standard‐of‐Care and Emerging Therapeutic Strategies

**DOI:** 10.1111/jgh.70354

**Published:** 2026-03-26

**Authors:** Landon L. Chan, Kallista O. K. Chan, Stephen L. Chan

**Affiliations:** ^1^ State Key Laboratory of Translational Oncology Hong Kong China; ^2^ Department of Clinical Oncology Sir YK Pao Centre for Cancer, Hong Kong Cancer Institute, Prince of Wales Hospital, The Chinese University of Hong Kong Hong Kong China

## Abstract

Hepatocellular carcinoma (HCC) is the most common type of primary liver cancer, accounting for up to 80% of all cases. Most patients present at an advanced stage and are unsuitable for curative treatments. In the past 5 years, immunotherapy combination has superseded tyrosine kinase inhibitors (TKI) as the standard first‐line therapy for advanced HCC. These therapies include the combination of anti‐PD‐(L)1 with an anti‐CTLA‐4 or partner with anti‐VEGF; these agents offer unprecedented high rates of response and survival. Unfortunately, only about 20%–30% of patients respond to first‐line immunotherapy combination, and about 50% of them would develop disease progression at 6 months. In patients with progression on immunotherapy, recent prospective studies support the efficacy and safety of multiple TKIs. To further improve the efficacy of systemic therapies, novel therapeutic strategies are actively being investigated, such as with the addition of a third immune checkpoint inhibitor. Furthermore, there is increasing interest to incorporate locoregional therapies in patients with advanced disease. Three Phase 3 randomized studies (EMERALD‐1, LEAP‐012, and TALENTACE) have recently demonstrated survival benefits with the combination of trans‐arterial chemoembolization (TACE) and immunotherapy, over TACE alone. The higher response rates brought by combining locoregional therapies and systemic therapies have enabled the possibility of downstaging and conversion. In addition, cellular therapy has shown promise in early phase studies, demonstrating potential to expand the use of immunotherapy in HCC beyond immune checkpoint inhibitors. In this review, we provide an overview of the current treatment landscape and emerging therapeutic strategies for advanced HCC.

## 1 Introduction

Liver cancer ranks third among causes of cancer‐related mortalities around the world, with approximately 800 000 deaths per year [1]. It is estimated by the WHO that by 2030, more than one million patients will die from liver cancer annually [1]. The most common primary liver cancer is hepatocellular carcinoma (HCC), accounting for up to 80% of all cases. HCC is closely linked to chronic infections such as hepatitis B and hepatitis C virus, alcohol‐associated liver disease (ALD), and metabolic dysfunction‐associated steatotic liver disease (MASLD) [2]. Although the adoption of vaccination programmes and antiviral therapies have reduced the incidence of viral HCC, the incidence of MASLD‐related HCC is increasing [2]. The expected global rise in HCC highlights the pressing need for effective systemic therapies to improve survival outcomes for patients with liver cancer.

For advanced HCC, immune checkpoint inhibitors (ICIs) are now the standard of care, reserving tyrosine kinase inhibitors (TKIs) for those who are unsuitable or contraindicated to ICIs [3]. These frontline ICIs combinations offer unprecedented high response rates and survival benefits to patients with advanced HCC, with an objective response rate (ORR) of 30% and a median overall survival (OS) reaching 2 years [4, 5, 6, 7]. Nevertheless, challenges remain as the most patients eventually develop disease progression and novel strategies are being actively explored to improve the clinical outcomes of patients with advanced HCC. These strategies include the addition of a third ICI to the existing regimens, incorporation of locoregional therapies, and exploration of cellular therapy. Furthermore, with longer survival observed with ICIs, it has become clinically relevant to discuss subsequent therapeutic strategies upon disease progression. In this expert review, we aim to discuss the current treatment landscape and the emerging therapeutic strategies that could improve treatment outcomes for advanced HCC.

## 2 Current Standard‐of‐Care

### 2.1 First‐Line Treatment

**FIGURE 1:**
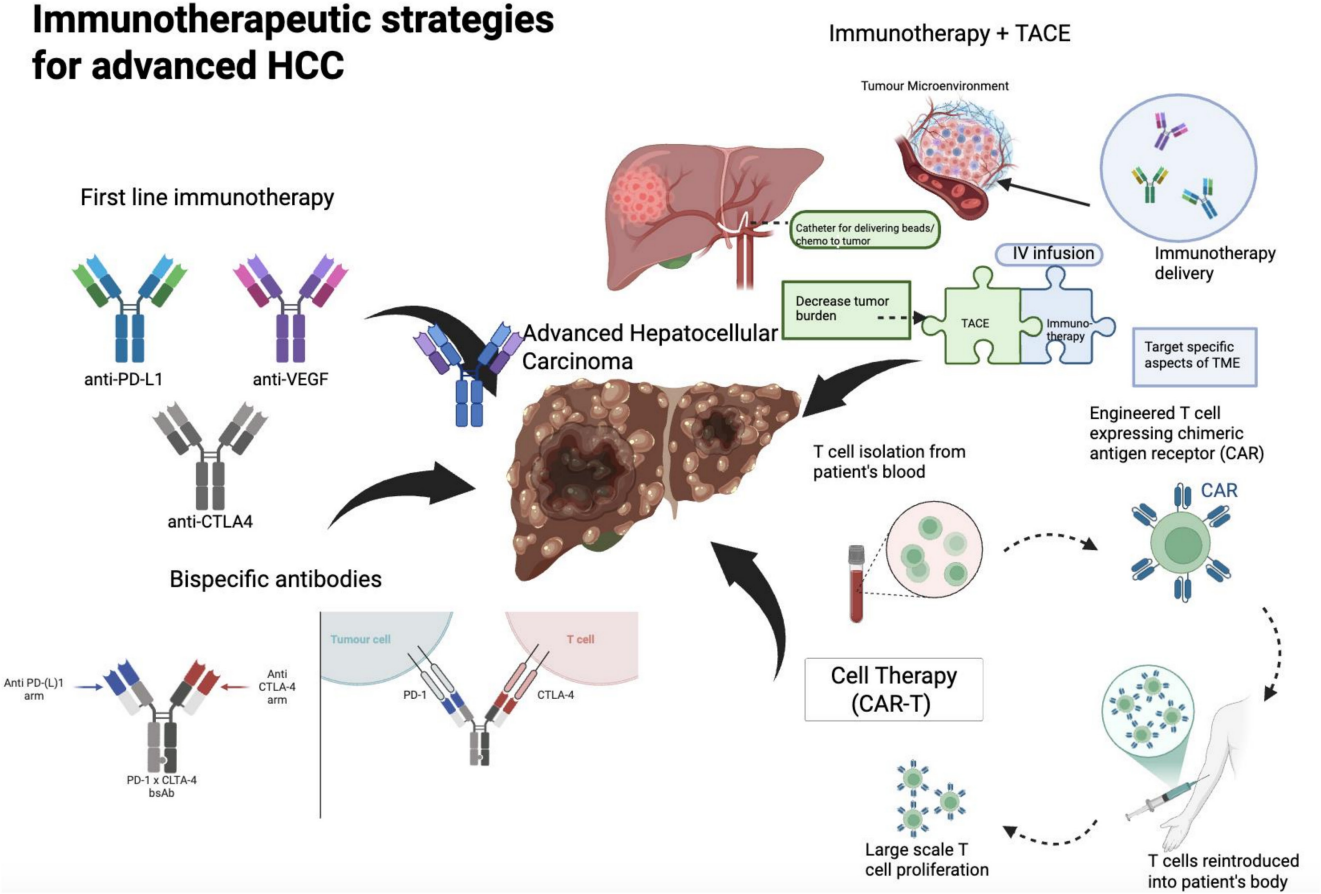
Immunotherapeutic strategies for advanced HCC involve combination immune checkpoint inhibitors. Combination of immunotherapy with locoregional therapy, such as TACE, has also shown improvement in survival. Novel immunotherapeutics, such as bispecific antibodies and CAR‐T cell therapy, are being actively explored.

Therapeutic landscape for advanced HCC has evolved rapidly over the years, demonstrating a remarkable shift from the use of TKIs to ICIs (Figure 1) [3]. The combination of atezolizumab, an anti‐PD‐L1 agent, plus bevacizumab, a vascular endothelial growth factor (VEGF) inhibitor, was the first ICI combination that showed superiority over TKI and is the current standard of care for first‐line treatment for advanced HCC [4, 8]. Such combination was evaluated in the IMbrave150 trial. The trial demonstrated a significant improvement in OS, PFS, and quality of life compared to sorafenib in patients with unresectable HCC. Atezolizumab plus bevacizumab achieved a median OS of 19.2 months, in comparison to the 13.4 months with sorafenib (HR 0.66, *p* < 0.001) (Table 1) [4].

**TABLE 1:** Approved immunotherapy combination regimens for advanced hepatocellular carcinoma.

	IMbrave150	HIMALAYA	Checkmate‐9DW
Study drugs	Atezolizumab plus bevacizumab	Tremelimumab plus durvalumab	Nivolumab plus ipilimumab
Mechanism of immunotherapy	Anti‐PD‐L1 and anti‐VEGF	Anti‐CTLA‐4 and anti‐PD‐L1	Anti‐PD‐1 and anti‐CTLA‐4
Number of patients evaluated in the experimental arm	336	388	335
Key inclusion criteria	Unresectable HCC, not suitable for locoregional therapy, Child–Pugh A; OGD within 6 months without high‐risk varices	Histological diagnosis of HCC, ineligible for curative therapy, Child–Pugh A; adequate endoscopic therapy for patients with history of EV bleed	Histological diagnosis of HCC, Child–Pugh A, not eligible for curative locoregional treatment; presence of portal hypertension with history of bleeding due to EV or GV within 6 months excluded
Allow for Vp4	Yes	No	No
ORR (%)	30	20	36
DCR (%)	74	60	68
Median PFS (months)	6.9	3.8	9.1
Median OS (months)	19.2	16.4	23.7
3‐year OS (%)	—	30.7	38
Grade ≥ 3 TRAEs (%)	43	25.8	41
TRAEs leading to death (%)	1.8	2.3	3.6
Use of high‐dose steroid (%)	12	20.1	29

Since the adoption of atezolizumab plus bevacizumab as the standard of care for advanced HCC, multiple studies have demonstrated its effectiveness in the real‐world setting in different subgroups of populations, highlighting this regimen's versatility and robustness. These include older patients [9], Chinese ethnicity [10], high‐risk and Vp4 (main portal vein invasion) cases [11], ALBI grade 1 [12], BCLC B [13], prior locoregional therapy [14], and nonviral etiology [15]. However, one concern with the use of atezolizumab plus bevacizumab is its bleeding risk. Anti‐VEGF agent such as bevacizumab is useful in targeting tumor cells via inhibition of angiogenesis. However, inhibition of VEGF also reduces the regenerative ability of endothelial cells and decreases their function, leading to higher bleeding risk [16]. Bevacizumab could delay the healing of esophageal variceal ligation (EVL) ulcers, which is normally promoted by VEGF via angiogenesis. Thus, treatment of atezolizumab plus bevacizumab requires endoscopic assessment to prophylactically treat any esophageal varices. In a recent meta‐analysis of 28 studies (involving 3895 patients), the pooled prevalence of bleeding side effects was 8.4%. About 4.4% of patients experienced serious (Grade 3 or 4) bleeding, and 2.1% of patients experienced fatal (Grade 5) bleeding [17]. These rates are similar to that reported in the IMbrave150 study and highlight the safety of this regimen in the real‐world setting.

Durvalumab plus tremelimumab is the second regimen approved for the treatment of advanced HCC. It consists of two ICIs, an anti‐PD‐L1 (durvalumab) and an anti‐CTLA‐4 (tremelimumab). The regimen was evaluated in the HIMALAYA trial, which demonstrated superior efficacy when compared to sorafenib, with a median OS of 16.4 months versus 13.8 months [5]. Notably, durvalumab plus tremelimumab was the first regimen to demonstrate durable survival benefits in patients with advanced HCC, with a 5‐year survival rate of 20% [18]. Grade ≥ 3 treatment‐related adverse events occurred in 26%, and 20% required high dose steroid due to immune‐related adverse events (irAEs). Nonetheless, only 8.2% of patients discontinued treatment due to toxicity, and 2.3% treatment‐related deaths were observed (Table 1).

Recently, the FDA approved the combination of nivolumab plus ipilimumab as another first‐line treatment for unresectable or metastatic HCC, which marks the expansion of the application of dual immune checkpoint blockade. In the Checkmate‐9DW study, nivolumab plus ipilimumab showed superior efficacy when compared to TKIs (15% sorafenib and 85% lenvatinib), with a median OS of 23.7 months versus 20.6 months (Table 1) [6]. Despite the promising survival outcomes with nivolumab plus ipilimumab, the early crossing of the Kaplan–Meier curves raised concerns about the safety and tolerability of this regimen. The pattern reflects a higher number of deaths during the first 6 months of treatment among patients receiving nivolumab plus ipilimumab when compared to those receiving TKIs. This high early mortality was primarily attributed to irAEs [6]. Importantly, in the nivolumab plus ipilimumab group, irAEs occurred in 58% of the treated patients, with 28% having Grade 3 to 4 events, and nearly 30% required high‐dose steroids. The high rates of fatal irAEs call for the application of this regimen in very fit patients and underscore the need for closer monitoring of patients on treatment.

Given multiple immunotherapy regimens are available as first‐line treatment for HCC, it has become relevant to decide the optimal treatment for each individual patient based on existing evidence, disease burden, and baseline characteristics. This is a complicated process because there is no head‐to‐head comparison between the approved regimens. Nonetheless, in a recent propensity‐score matching analysis of 618 patients, it demonstrated similar OS (approximately 15–16 months) between durvalumab plus tremelimumab and atezolizumab plus bevacizumab [19].

In addition to immunotherapy, TKIs are still one of the cornerstones of systemic therapy for advanced HCC. Although immunotherapy is more commonly preferred nowadays, when immunotherapy is contraindicated or unavailable, TKIs are a reasonable treatment option. These include lenvatinib, which is the preferred option, and sorafenib if lenvatinib is not available. Indeed, in modern cohorts, the median OS of lenvatinib can reach up to 19 months [20], which is numerically similar to atezolizumab plus bevacizumab in the IMBrave150 study.

### 2.2 Second‐Line Treatment

Although first‐line immunotherapy for advanced HCC has brought unprecedented survival benefits, across the major trials examining immunotherapy combination in advanced HCC, only about 30% of patients respond to treatment, and the median PFS was only about 6 months [21]. Resistance to immunotherapy in HCC is complex and incompletely understood. Several mechanisms have been postulated to confer resistance to immunotherapy. These resistance mechanisms can be broadly classified into intrinsic or extrinsic [22]. Intrinsic mechanisms, which refer to pathways within the tumor itself, include activating mutations along the Wnt/β‐catenin pathway [23], defective TP53 [24], and alterations in the interferon‐gamma signaling pathway or antigen presenting machinery [25]. Extrinsic mechanisms, which relate more to the tumor microenvironment, include the abundance of exhausted T cells [26], the absence of effective T‐cell infiltrates [27], activation of hepatic stellate cells [28], and modulation by the gut microbiome [29, 30].

Following progression on first‐line immunotherapy, about 40%–50% of patients will be eligible for further systemic therapies [21]. According to the latest international guidelines, there are no standard treatment recommendations for the second‐line therapy. However, prospective studies support the use of TKIs, including regorafenib [31], cabozantinib [32], and lenvatinib [33]. REGONEXT trial, a phase II prospective study, evaluated safety and effectiveness of regorafenib in unresectable HCC patients who progressed on the first‐line atezolizumab plus bevacizumab. Forty patients were enrolled in this study (Table 2). The median PFS was 3.5 months, and the median OS was 10.5 months. ORR and DCR were 10.0% and 82.5%, respectively. These results are comparable to the data demonstrated in the RESOURCE trial (median PFS 3.1 months and median OS 10.6 months), in which patients received sorafenib as first‐line treatment [34]. Another multicenter Phase II trial investigated cabozantinib among patients with advanced HCC, who have progressed after prior ICI‐based therapy [32]. In this study, patients treated with cabozantinib achieved an ORR of 6.4%, a median PFS of 4.3 months, and OS of 14.3 months (Table 2) [32]. Again, these results are similar to the CELESTIAL study when cabozantinib was used as a second‐line treatment for patients who progressed on sorafenib [35]. For lenvatinib, a recently published multicenter Phase II study conducted in South Korea showed that lenvatinib demonstrated meaningful clinical activity as a second‐line treatment post atezolizumab plus bevacizumab (Table 2). The ORR was 12%, median PFS 5.4 months, and a median OS was 9.8 months [33].

**TABLE 2:** Efficacy and safety of TKIs following prior first‐line immunotherapy.

	Cabozantinib	Lenvatinib	Regorafenib
Study (year)	Chan et al. (2024)	Kim et al. (2025)	Cheong et al. (2025)
Number of patients	47	50	40
Prior first‐line atezo‐bev (%)	55.6	100	100
ORR (%)	6.4	14.0	10.0
DCR (%)	76.6	82.0	82.5
Median PFS (months)	4.1	5.4	3.5
Median OS (months)	9.9	9.8	10.5
Most common TRAEs	PPE (42.6%), fatigue (38.3%), diarrhea (21.2%), proteinuria (21.2%)	Diarrhea (42.0%), hypothyroidism (32.0%), anorexia (30.0%)	PPE (70.0%), anorexia (25.0%), fatigue (22.5%), hyperbilirubinemia (22.5%)
Most common grade ≥ 3 TRAEs (%)	Thrombocytopenia (6.4%), hypertension (4.3%)	ALT increase (10.0%), hypertension (8.0%), proteinuria (8.0%)	Thrombocytopenia (2.5%)

Abbreviations: ALT, alanine aminotransferase; Atezo‐bev, atezolizumab and bevacizumab; DCR, disease control rate; ORR, objective response rate; OS, overall survival; PFS, progression‐free survival; PPE, palmar‐plantar erythrodysesthesia; TRAEs, treatment‐related adverse events.

In the setting of disease progression on immunotherapy, rechallenge with an anti‐CTLA‐4 represents another viable strategy. It provides an alternative antitumor mechanism and may theoretically enhance the priming of antitumor immunity. This approach has been evaluated across multiple cancer types, with higher efficacy reported in melanoma but more variable results in others, such as renal cell carcinoma and non–small‐cell lung cancer [36]. In HCC, small‐scale studies have reported modest efficacy and acceptable safety for anti‐CTLA‐4 rechallenge in patients progressing on prior anti‐PD‐(L)1 (with or without anti‐CTLA‐4 agent), or atezolizumab plus bevacizumab. For instance, in the HIMALAYA trial, approximately 8% of patients received a second dose of tremelimumab upon disease progression [5]. Although efficacy outcomes for this rechallenge have not yet been fully reported, it has been shown to be safe, with only 10% of the tremelimumab‐rechallenged population experiencing immune‐mediated adverse events that require high‐dose steroids [37].

Retrospective studies have also reported efficacy outcomes associated with anti‐CTLA‐4 rechallenge. For example, a small case series of 10 patients with prior progression on immunotherapy (seven of whom progressed on atezolizumab plus bevacizumab) reported rechallenge with nivolumab plus ipilimumab provided an ORR of 30%, disease control rate of 40% and a median PFS of 2.9 months [38]. In an international, retrospective, multicenter study exploring the efficacy of immunotherapy rechallenge, among the eight patients who received subsequent nivolumab plus ipilimumab in patients who progressed on atezolizumab plus bevacizumab, two achieved an objective response and one additional patient achieved stable disease [39]. Another prospective study from Japan reported an ORR of 5% and a median PFS of 2.9 months in patients who received second‐ or later‐line durvalumab plus tremelimumab in patients with prior atezolizumab plus bevacizumab [40].

Overall, while these data suggest potential clinical activity with rechallenge of anti–CTLA‐4 after progression on prior immunotherapy, responses remain modest and limited by small sample sizes. Further prospective evaluation is warranted to better define its role in this setting.

## 3 Emerging Therapeutic Strategies in Advanced HCC

While current first‐line and second‐line treatments for advanced HCC have shown significant improvements in treatment, they are not without limitations. One important challenge is to address primary resistance and secondary resistance to treatment. More than half of the patients with advanced HCC do not derive a response to first‐line immunotherapy, and the majority of patients would eventually develop disease progression. This highlights an unmet clinical need to improve response and treatment outcomes with existing therapeutics. To that end, synergism between systemic and locoregional therapies and novel immunotherapeutics is actively being investigated.

### 3.1 Synergistic Approaches: Combining Immunotherapy and Locoregional Therapy

HCC is highly heterogenous with various molecular alterations and a multifaceted tumor microenvironment, yet there are no one single actionable mutations thus far, which could explain the general ineffectiveness of monotherapies [41]. Furthermore, immunotherapy combinations typically only offer response rates of about 20%–30%, which indicate the absence of tumor antigenicity and the presence of an immunosuppressive tumor microenvironment in HCC [41]. The combination of locoregional therapy can destroy the primary tumors, decrease tumor burden, and induce the exposure of tumor antigens, which could potentially enhance the effectiveness of immunotherapy without overlapping toxicities (Figure 1) [42].

The combination of immunotherapy with locoregional treatments has been a focus of active research in the management of HCC, targeting patients with unresectable HCC. The EMERALD‐1 study is the first phase III trial designed to evaluate the combination of durvalumab, bevacizumab, and TACE versus TACE alone in patients with embolization‐eligible unresectable HCC [43]. The study met its primary endpoint, demonstrating improvement in PFS, with median PFS of 15.0 months in the combination arm when compared to 8.2 months in TACE‐alone arm (HR 0.77, *p* = 0.032). An absolute 15% increase in ORR was also observed in the combination arm. Adverse events were manageable and consistent with the known profiles of individual agents. Grade 3 or higher treatment‐related adverse events occurred in 27% in the combination arm, most commonly related to anti‐VEGF side effects such as hypertension and proteinuria [43]. Similarly, the LEAP‐012 is a Phase III trial evaluating the efficacy and safety of combing TACE and immunotherapy. Patients were assigned to receive pembrolizumab plus lenvatinib combined with TACE, or TACE alone. A significant improvement in median PFS was again observed, with a median PFS of 14.6 months in the combination arm, compared to 10.0 months only in the TACE alone arm (HR 0.66, *p* = 0.002) [44]. ORR was also improved in the combination arm, at 47% compared to 33% in the TACE alone arm. In terms of safety, Grade 3 or higher treatment‐related adverse events occurred in 71% of patients in the lenvatinib plus pembrolizumab group, with key toxicities including hypertension and thrombocytopenia. TALENTACE is a Phase III study conducted mainly in China and Japan, which evaluated efficacy and safety of combining on‐demand TACE and atezolizumab plus bevacizumab compared to TACE alone. In contrast to EMERALD‐1 and LEAP‐012 which enrolled any tumor burdens, TALENTACE enrolled patients with higher tumor burden (e.g., sum of tumor maximum diameter plus tumor number larger than 6) [45]. The primary endpoint is investigator assessed TACE‐PFS, which is defined as the time from randomization to untreatable progression or TACE failure/refractoriness, or death by any cause [45]. The study again showed that combination treatment improved TACE‐PFS,at 11.3 months versus 7.0 months with TACE alone [45]. ORR by RECIST 1.1 was 49.1% in the combination arm, compared to 33.9% in the TACE alone. There were no new safety concerns with the combination treatment. Given the results of these Phase 3 studies demonstrating improvement in PFS with combination therapy, these regimens have been incorporated in the latest international guidelines, setting a new standard for unresectable HCC [3].

The promising treatment outcomes for unresectable HCC brought by combining immunotherapy and locoregional therapies have introduced the concept of downstaging and conversion. The ABC conversion therapy study evaluated atezolizumab plus bevacizumab followed by curative conversion (including resection, ablation, and transarterial chemoembolization [TACE]) in 110 intermediate‐stage, TACE‐unsuitable HCC patients (Child–Pugh A) [46]. With this strategy, 38 patients (35%) received curative conversion due to response to treatment. Notably, 25 of them attained drug‐free status, which required the disease to be completely resected, tumor markers being normalized for 24 weeks, and complete response on contrast enhanced ultrasound [46]. Among patients who received conversion treatment and achieved drug‐free status, none of these patients relapsed after 3 years. These results reflected how combination treatments could be utilized to achieve long‐term remission in patients with initially incurable disease.

### 3.2 Novel Immunotherapeutic Strategies

To improve response and survival of first‐line immunotherapy, several approaches have been adopted. They include addition of a third ICI to enhance the efficacy of immunotherapy, development of cell therapy to release a stronger antitumor immunity, and building bispecific antibodies that could reduce peripheral toxicities.

TIGIT is an immune checkpoint receptor expressed on CD8+ T and CD4+ T cells, NK cells, and Tregs, which acts as an inhibitory receptor to suppress both adaptive and innate immunity [47]. TIGIT could act as a compensatory immune checkpoint when PD‐1 blockade occurs [47]. Based on this principle, anti‐TIGIT was first evaluated for HCC in the Phase Ib/II Morpheus‐Liver study [48]. In this study, patients with advanced HCC was randomized in a 2:1 fashion to receive atezolizumab, bevacizumab, and tiragolumab (anti‐TIGIT) versus atezolizumab plus bevacizumab. Among the 40 patients who were randomized to the tiragolumab arm, a motivating signal was observed with an ORR of 42.5%. The median PFS was 12.3 months, and the median OS was 28.9 months. In the atezolizumab plus bevacizumab control group (*n* = 18), the ORR was only 11%, median PFS was 4.2 months, and median OS was 15.1 months [48]. Building upon the foundation of the Morpheus‐Liver study, IMbrave152 trial was designed to evaluate whether addition of tiragolumab to atezolizumab plus bevacizumab could improve PRS and OS in patients with advanced HCC. Unfortunately, at the ESMO annual conference 2025, it was shown that the triplet regimen did not improve ORR (29.9% versus 26.0%) and PFS (8.3 months versus 8.2 months, *p* = 0.75) [49].

It is not entirely clear why tiragolumab failed to demonstrate additional benefit with atezolizumab plus bevacizumab. One reason could be the number of patients in the control arm of Morpheus‐Liver was too small (*n* = 18) to be generalized to the large heterogenous population in the real world, not to mention the performance of the control arm was markedly worse than the IMBrave150 study, with a significantly lower response rate and PFS. The proceeding to a randomized Phase 3 study based on Morpheus‐Liver could has been optimistic. Another reason is probably that tiragolumab is not an active oncological drug as it has failed almost completely in the entire SKYSCRAPER studies. These highlights that a larger sample size might be needed to derive better estimates on the true difference between the experimental arm and control arm even in earlier phase studies.

Another study that examined the addition of a third ICI is the TRIPLET‐HCC study. It is a Phase 2/3 randomized trial assessing the effectiveness of the addition of low‐dose ipilimumab (1 mg/kg) to atezolizumab plus bevacizumab, compared to atezolizumab plus bevacizumab. This is the first randomized study that utilized atezolizumab plus bevacizumab as the control arm. Preliminary safety data suggest that the triple combination therapy has a manageable safety profile without unexpected toxicities, supporting further investigation into its efficacy and long‐term safety [50]. However, in the ESMO annual conference 2025, it was announced that there was no difference in ORR (triplet: 30.1% versus doublet: 27.4%), PFS (8.0 months versus 9.6 months), and OS (22.6 months versus not reached), when low‐dose ipilimumab (1 mg/kg) was added, but a modest degree of increased toxicity was observed in the triplet arm (grade ≥ 3 treatment‐related toxicities: 44% versus 39%; 6 versus 0 deaths) [51].

Chimeric antigen receptors T cell therapy (CAR‐T) is a novel immunotherapy that involves synthetically engineering antigen receptors on T cells to recognize tumor antigens to enhance antitumor immunity (Figure 1) [52]. One advantage of CAR T cells is that they can bind directly to surface antigens without antigen presentation via major histocompatibility complex (MHC) molecules, thereby enabling a prompt and effective antitumor response [53]. CAR‐T therapies have demonstrated effectiveness in treating patients with refractory lymphoma and are recommended in international guidelines [54]. However, progress in its adoption in solid tumors has been slow due to difficulties in identifying target and off‐target toxicity.

Recently, the development of GPC3‐targeted CAR‐T has shown great promise. GPC3 is a protein found on the placenta which plays a role in morphogenesis [55]. The expression of GPC3 in adults is limited to a few tissues, and there is only limited expression in cirrhotic liver, making it an attractive target for cell therapy development [56] In a Phase I clinical trial that evaluated the safety and preliminary efficacy of C‐CAR031, a GPC3‐targeted, TCF‐β‐resistant CAR‐T in 24 heavily pretreated patients with advanced HCC, no dose‐limiting toxicity or ICANS occurred, indicating a manageable safety profile. Of interest, 90.9% achieved tumor reduction, with an ORR of 57.1% at the highest dose. Even though 91.7% developed cytokine release syndrome (CRS), all of which resolved. No immune effector cell‐associated neurotoxicity syndrome (ICANS) was observed.

This demonstrated encouraging antitumor activity of C‐CAR031 in heavily treated advanced HCC patients, who typically have a limited number of treatment options available.

Bispecific antibodies (BsAbs) are designed to engage two distinct antigens or pathways, therefore offering several advantages over monoclonal antibodies, including overcoming tumor heterogeneity and limiting peripheral toxicity [57]. Cadonilimab is a BsAb that simultaneously targets both PD‐1 and CTLA‐4. Its Fc‐null design contributes to lower toxicities and enhanced antitumor activity [58]. It was evaluated in the COMPASSION‐08 Phase Ib/II clinical study in two dose levels [59]. In the lower dose cohort (6 mg/kg every 2 weeks, *n* = 31), the combination demonstrated an ORR of 35.5%, median PFS of 8.6 months, and median OS of 27.1 months. In the higher dose cohort (15 mg/kg every 3 weeks, *n* = 28), the ORR was 35.7%, median PFS was 9.8 months, and the median OS was not reached. GEMINI‐Hepatobiliary is a multidrug, master protocol, Phase 2 study that evaluates the safety and efficacy of a novel BsAb volrustomig (anti‐PD‐1/CTLA‐4) monotherapy or in combination with bevacizumab or lenvatinib [60]. It has completed recruitment, and the results from the HCC cohort are expected to be announced next year.

### 3.3 Current and Emerging Biomarkers for Development

Currently, alpha‐fetoprotein (AFP) remains the only widely adopted biomarker in HCC. AFP is expressed by the majority of HCC tumors and correlates with pathological grade, prognosis, and treatment response [61]. However, its utility in selecting patients for specific therapies has not been clearly demonstrated and has only been adopted in one Phase 3 trial. In the REACH‐2 study, patients with advanced HCC who had progressed on sorafenib and had elevated levels of AFP (≥ 400 ng/mL) were randomized to receive either ramucirumab or placebo [62]. In this biomarker‐selected population, ramucirumab achieved a modest improvement of 1.2 months in both PFS and OS. Nevertheless, in the current era of immunotherapy, contemporary regimens have shown comparable efficacy in both AFP‐expressing and nonexpressing patients [5, 6, 10].

Targeting fibroblast growth factor receptor 4 (FGFR4) represents an emerging strategy for biomarker‐driven therapy in HCC. FGF19 is overexpressed in approximately 30% of HCC cases, where it binds to FGFR4 to regulate bile acid metabolism and hepatocyte proliferation [63]. Aberrant activation of the FGF19‐FGFR4 signaling pathway has been implicated in hepatocarcinogenesis. Early phase studies have shown that FGFR4 inhibitors demonstrated ORRs of 15%–20% in patients with prior treatments [64, 65]. Despite these initial promises, these agents were not further developed due to the rapid advancements in immunotherapy at the same time. More recently, however, a Phase 2 study in an FGF19‐enriched population evaluating the selective FGFR4 inhibitor irpagratinib in combination with atezolizumab reported an ORR of 51.7% and a median PFS of 7.0 months [66]. As a result, a prospective Phase 2 study has been proposed to assess the triplet regimen of irpagratinib, atezolizumab, and bevacizumab in patients with unresectable HCC (NCT07010497). The results of this study are eagerly awaited to determine whether a biomarker‐selected approach can further improve response rates and survival in advanced HCC.

Immune signatures have also been investigated as biomarkers to predict immunotherapy outcomes. The immune contexture of HCC has been classified into two distinct subclasses: the immune‐inflamed class (approximately 35% of tumors) and the noninflamed class (approximately 65% of tumors) [27]. The inflamed class is characterized by enrichment of CD8+ T cell and M1 macrophages infiltration. This phenotype is associated with favorable responses to immunotherapy. In contrast, the noninflamed subclass features a predominance of regulatory T cells (Tregs) and M2 macrophages, along with frequent mutations in CTNNB1 and TP53 mutations in the tumor. These tumors generally are resistant to immunotherapy [24]. Independently, an integrated molecular analysis of 358 tumor samples from the GO30140 Phase Ib and IMbrave150 study, of patients who were treated with atezolizumab plus bevacizumab, identified that high preexisting immunity such as high expression of CD274 and intratumoural CD8+ T cell density, was associated with better clinical outcomes. Conversely, less clinical benefit was associated with high Tregs to effector T cell ratio and expression of oncofetal genes such as GPC3 and AFP [67]. Putting together, these findings suggest that a lack of infiltrative effector T cells in the tumor microenvironment contributes to diminished immunotherapy response. Nevertheless, routine clinical implementation of these immune signatures remains limited, especially as many HCC cases are diagnosed radiologically without biopsy.

Lastly, etiology‐specific differences in response to immunotherapy have been proposed. It has been postulated that patients with nonviral etiology would respond poorly to immunotherapy, whereas higher benefits were observed for patients with viral etiology. This is supported by a preclinical NASH mouse model showing potential detrimental effect of anti‐PD‐1 therapy with accelerated carcinogenesis and auto‐aggressiveness [68]. However, two recent meta‐analyses including modern clinical trials comparing immunotherapy with TKIs concluded that immunotherapy provides benefit across etiologies [69, 70]. Although hazard ratios were numerically lower in viral HCC, the differences between viral and nonviral HCC were not statistically significant. Furthermore, nonviral etiology encompasses a heterogeneous group, including metabolic‐associated steatotic liver disease (MAFLD)‐related HCC and alcoholic liver disease (ALD)‐related HCC. It remains unclear whether specific subgroup analyses from the COSMIC‐312 trial suggested reduced benefit in NASH‐HCC compared with ALD‐HCC [71], whereas post hoc analysis of IMBrave150 indicated numerically lower response rates and survival in ALD‐HCC relative to viral HCC and NASH‐HCC [72]. These observations highlight the complexity of using etiology as a biomarker to predict outcomes to immunotherapy. Until further studies are being conducted to compare immunotherapy efficacy across distinct etiologies, immunotherapy should be considered beneficial to all patients with advanced HCC.

## 4 Conclusion and Future Directions

Systemic therapy for HCC in 2025 witnessed progresses and challenges. The approval of nivolumab plus ipilimumab based on the results of Checkmate‐9DW study has complicated the choice of first immunotherapy in advanced HCC. Given the high ORR and survival achieved with nivolumab plus ipilimumab, this regimen could be reserved for those who are considered fit, taking into account the high risk of irAEs and potential treatment‐related mortality. Atezolizumab plus bevacizumab remains a versatile option with strong real‐world data to support its use in the majority of patients with advanced HCC, except for those with clear contraindication to anti‐VEGF. However, the lack of long‐term data and the potential of damage to liver function with prolonged use of anti‐VEGF in cirrhotic patients remain a limitation. The combination of durvalumab plus tremelimumab is highly appealing due to its robust long‐term data and favorable side effect profile. Emerging real‐world evidence further supports its use. However, the regimen's lower response rate and absence of biomarkers to identify patients most likely to benefit warrant careful consideration. The recent failure of IMBrave152 and the TRIPLET study demonstrated that the addition of a third ICI in a biomarker unselected population is not the way to go forward.

As we wait for novel therapeutics to further improve outcomes for patients with HCC, several directions have shown promise. First, the combination of locoregional therapies with systemic therapy has shown improved response and survival. Notably, some patients initially thought to have advanced, unresectable disease were converted to cure with this approach. Identifying these patients suitable for this approach in the multidisciplinary meeting would be important. Second is the emerging novel immunotherapeutics such as CAR T therapy and bispecific antibodies. However, large‐scale studies and experience in handling CRS and ICANS would be required to define the efficacy and safety of these new agents.

In conclusion, 2025 has been an eventful year for HCC, marked with progresses and challenges. It is anticipated that an increasing number of treatment strategies will become available in the future as patients with advanced HCC liver longer. Treatment sequencing in this evolving landscape will be relevant in the next decade.

## Conflicts of Interest

S.L.C. serves as an advisory member for AstraZeneca, MSD, Eisai, BMS, Ipsen, and Hengrui and received research funds (to institution) from MSD, Eisai, Ipsen, SIRTEX, and Zailab and honoraria from AstraZeneca, Eisai, Roche, Ipsen, and MSD. L.L.C. has received research funding (to institution) from Roche, advisory board member for Roche, honoraria, and travel support from AstraZeneca, Eisai, Ipsen, and Roche.
